# Association of arginase I or nitric oxide-related factors with job strain in healthy workers

**DOI:** 10.1371/journal.pone.0175696

**Published:** 2017-04-12

**Authors:** Keiki Ogino, Tatsuo Ito, Eri Eguchi, Kenjiro Nagaoka

**Affiliations:** Department of Public Health, Okayama University, Graduate School of Medicine, Dentistry and Pharmaceutical Sciences, Okayama, Japan; Boston University, UNITED STATES

## Abstract

This study evaluated the associations between job strain and arginase I in 378 healthy Japanese factory workers by a cross-sectional study measuring nitric oxide (NO)-related parameters (arginase I, L-arginine, exhaled nitric oxide (FeNO), and NOx), clinical parameters, and job strain using a Japanese version of the Job Content Questionnaire by Karasek. Arginase I and FEV1% were negatively correlated with job strain and positively correlated with job control and social support. FeNO and hs-CRP were negatively correlated with job strain. Multiple regression analysis showed negative association of arginase I with job strain and positive association with job control and social support in females. It is concluded that serum levels of arginase I may be useful biomarkers for the diagnosis of job stress in healthy female workers, although many factors can be influencing the data.

## Introduction

Overwork-related stress has an effect on human health. In particular, sleep disturbance is the main complaint in work stress caused by overworking. Moreover, sleep disturbance evokes psychological disorders such as depression. Severe depression often leads to suicide. Changes in the industrial structure due to IT development has led to an increase in the number of workers with depression. Therefore, mental stress in the workplace has become a social problem in which the government should intervene in Japan.

Work stress is also known to be a risk factor for cardiovascular disease and its underlying factors, hypertension and atherosclerosis [1, 2]. Within the field of research on work stress, the demand-control model established by Karasek et al. [3] is well known; this model predicts that biological aversive strain will occur when the psychological demands of the job exceed the resources for control over task content. This combination of high demands and low control produces job strain. Workers in high job strain have been shown to have greater risk for developing cardiovascular disease. [2, 4, 5]. Although this work stress model is used for evaluation of the progression of atherosclerosis and cardiovascular disease, the underlying mechanism is not clear.

On the other hand, nitric oxide (NO) is generated from three types of NO synthase (NOS-1, 2, 3). NO from NOS-1 in neuron cells acts as a neurotransmitter and modulates other neurotransmitters, such as norepinephrine, serotonin, dopamine, and glutamate, and may be associated with depression [6, 7]. In exhalation, NO is measurable and plays an important role in the diagnosis of airway inflammation conditions such as asthma [8]. Fractional exhaled NO (FeNO) showed down-regulation in depression [9, 10]. Moreover, arginase, a key enzyme in the urea cycle, is involved in indirect regulation of NO by the consumption of L-arginine, which is a common substrate for NOS. Studies on the induction or activation of arginase have focused on pre-atherosclerotic vascular changes and asthmatic airway inflammation as pathophysiological evidence that the consumption of L-arginine by arginase may lead to the reduction of NO, resulting in the reduced enlargement of endothelial or bronchial smooth muscle-associated vascular damage and asthma [11].

Serum arginase levels were evaluated in various diseases, such as asthma [12, 13], sickle cell disease-induced asthma, pulmonary hypertension [14], cancer [15], and psychological depression [16] by activity assay and the ELISA method. In a healthy population, arginase I was associated with oxidative stress, exhaled nitric oxide, and L-arginine [17–19].

Although there is considerable evidence showing the association of depression and NO, there are no reports demonstrating the association of work stress or psychological stress with arginase. Therefore, in this study, we evaluated the interaction of arginase I with work stress in association with NO-related factors in healthy individuals of occupational workers.

## Material and methods

### Study design

**Fig 1:**
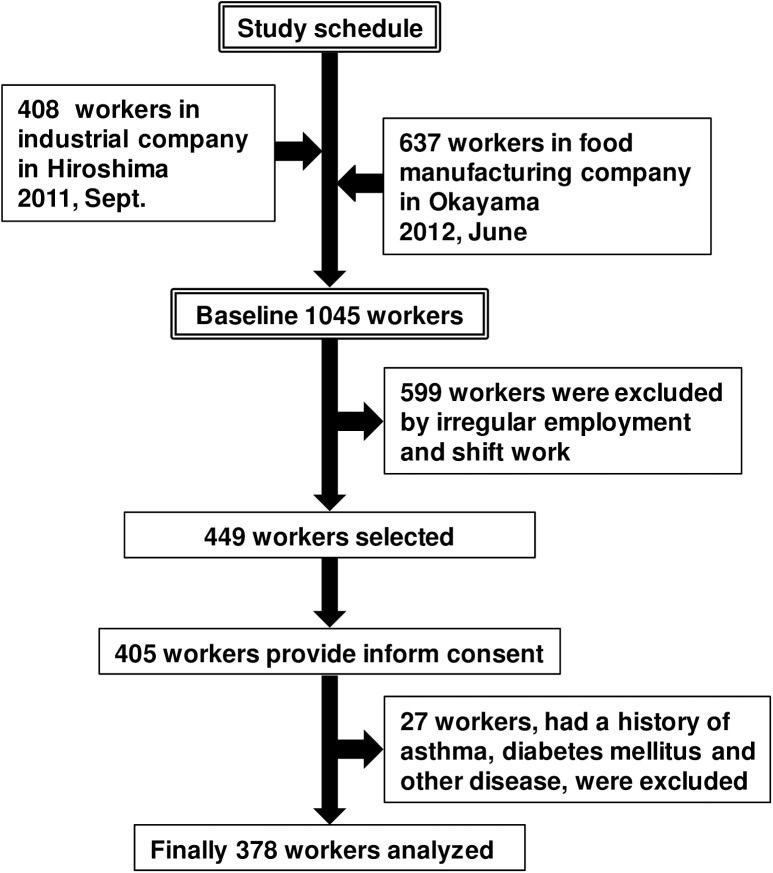
Flow chart of the study design from the two companies.

A cross-sectional study on the relationship between NO-related factors and job stress was designed within a workplace field study. Among all employees (n = 1045) in an industrial company (n = 408) in Hiroshima Prefecture in September 2011 and a food manufacturing company in Okayama Prefecture (n = 637) in June 2012, 599 individuals were excluded due to irregular employment and shift work, and then 449 were selected as candidates for this survey. Finally, 405 employees provided informed consent (written), and the data of 378 of those who had no previous history of asthma, diabetes mellitus, or other serious disease were used for the analysis. We performed this population study for two or three days in the morning from 9:00 to 12:00. A flow chart of study design is shown in Fig 1. The ethics committee of Okayama University approved the study (Number 561).

### Assessment of job strain

For assessment of work-related stress, we employed self-administered questionnaires including the 22-item Japanese version of the Job Content Questionnaire (JCQ) [20–22]. The JCQ model concerns job demands (5 items), job control (9 items), and worksite social support (8 items), with four-point response potions from 1 (strongly disagree) to 4 (strongly agree). The reliability of validity of the JCQ has been reported to be excellent for assessing job stress among Japanese employee [21, 22]. Question items for job demands were the speed in completing work, the degree of difficulty of the work, the amount of work, and the time allowed to complete the work and conflicting demands. The job control scale is a linear composite that measures decision-making authority, task variety, and personal freedom on the job. Social support includes items to evaluate support from supervisors and co-workers. Job strain was calculated as the ratio of the score of crude job demand to the score for crude job control. Then, the participants at the median value or higher ratio were indicated to be high strain.

### Serum subclinical parameters

Venous blood samples were collected, and sera were preserved at -80ºC until analysis. Serum high-sensitivity CRP (hs-CRP) was measured by a highly sensitive CRP assay (Behring Latex-Enhanced using the Behring Nephelometer BN-100; Behring Diagnostics, Westwood, MA, USA). Arginase I was determined using ELISA kits of Bethyl Laboratories (Montgomery, TX, USA) and Hycult Biotechnology b.v. UDEN, respectively. Information on lifestyle factors, including cigarette smoking, past medical history, and present steroid medication, was obtained using self-reported questionnaires or clinical records.

### Fractional exhaled NO (FeNO) and FEV1%

Fractional exhaled NO (FeNO) was measured using a portable electrochemical analyzer (Niox Mino, Aerocine AB, Sweden). This device measures FeNO during a 10-s exhalation with a constant flow of 50 mL.s^-1^, according to the international recommendations. All measurements were performed in duplicate, all within 10% deviation, and the mean concentration in parts per billion (ppb) was registered.

FEV1% was measured as a pulmonary function test using a spirometer (CHESTGRAPH Jr.; Chest, Tokyo, Japan) according to international recommendations, measuring FEV1 and FVC, and expressed as a percentage of FEV1/FVC.

### Serum NOx and L-arginine

NOx (NO_2_^-^ + NO_3_^-^) levels in the serum were determined with a NO analyzer (model-280i NOA with the Purge Vessel; Sievers, Boulder, CO, USA) to evaluate the correlation of arginase I with nitric oxide (NO) generation [23]. Serum was treated with nitrate reductase (Sigma-Aldrich, St. Louis, MO, USA) for 30 min at room temperature to convert nitrate to nitrite. Nitrite was further reduced to NO in the Purge Vessel containing the reducing agent potassium iodide in acetic acid, and NO was subsequently detected by the ozone-chemiluminescence method. Serum L-arginine was measured using an HPLC system (HITACHI, Tokyo, Japan) [24]. L-Arginine was eluted from serum and supplemented with 5 μM monomethylarginine (MMA) as an internal standard, using Oasis MCX solid phase-extraction cartridges (Waters, Milford, MA. USA) conditioned with 2 mL of methanol/water/ammonia solution (50:45:5, vol/vol/vol) and phosphate-buffered saline (PBS). Serum samples containing 5 μM MMA were dissolved in PBS and loaded on an equilibrated SPE column. The column was constitutively washed with 0.1 N HCl (2 mL) and methanol (2 mL). The fraction containing L-arginine was eluted with 1 mL of methanol/water/ammonia solution (50:45:5, vol/vol/vol) and dried in a vacuum centrifuge. After the drying process, the residue was reconstituted with water and mixed with an equal amount of derivatizing agent (5 mg/mL ortho-phthaldialdehyde, 10% methanol, 0.5% 3-mercaptopropionic acid in 200 mM borate buffer, pH 8.5), and the reaction was allowed to occur for 30 min at room temperature. The sample was introduced into the fluorescence HPLC system using a TSKgel ODS-100V column (4.6 x 250 mm, 5 mm, Tosoh, Yamaguchi, Japan). The mobile phase consisted of 9% acetonitrile in acetate buffer (pH 6.3) at a flow rate of 1.5 mL/min, and the excitation and emission wavelengths were 340 and 455 nm, respectively.

### Statistical analysis

Statistical analyses were performed using GraphPad Prism 5.0c for Mac (GraphPad Software, Inc., San Diego, CA, USA) and PASW Statistics 18 for Mac. The differences in means ± SE of several clinical parameters and job strain between sex, age (< 44 and ≥ 45), and companies were analyzed with the Mann-Whitney U test or unpaired *t* test and age-, sex-, and company-adjusted ANCOVA. The relationships between job strain and NO-related parameters or several clinical parameters were analyzed by Spearman’s correlation using the natural logarithm-transformed values. For comparisons of the proportions of smokers, alcohol drinkers, and those with exercise habits, chi-square test was used. When the values of job strain were divided into quartiles, differences in mean values of several NO-related parameters and other clinical parameters were analyzed by age-, sex-, and company-adjusted ANCOVA. A multiple regression analysis was performed to investigate the association of arginase I with job strain, job demand, job control, and social support. Covariate for adjustment were log-transformed values of age, sex, BMI, L-arginine, NOx, FeNO, FEV1%, systolic blood pressure, and hs-CRP, and dichotomized scale variable of smoking habit, exercise, and alcohol consumption. A value of P < 0.05 was considered to be significant.

## Results

### Characteristics of subjects by several clinical parameters

The basic characteristics of the subjects by several clinical parameters and job strain in this study are presented in Table 1 and Table 2. According to NO-related clinical parameters, high serum levels were observed for arginase I and L-arginine in males compared with those in females. In contrast, the NOx level was high in those aged over 45 compared with that in those aged under 44. Job strain was high in females, and job control was high in males. The rates of smoking, alcohol drinking, and exercise were high in males (Table 1).

**Table 1:** Caracteristics of subjects by several clinical parameters, job strain and life styles.

Variables	Total			Male			Female			P	Age < 44			Age ≥ 45			P
	(n = 378)			(n = 192)			(n = 186)				(n = 190)			(n = 188)			
Age	44.1	±	12.7	43.4	±	12.8	44.7	±	12.7	0.266	33.4	±	7.4	54.9	±	6.2	**< 0.0001**
Sex (M/F)	192	/	186								100	/	90	92	/	96	0.472
Company (1/2)	160	/	218	99	/	93	61	/	125	**0.0002**	67	/	123	93	/	85	**0.001**
BMI	23.1	±	3.6	24.0	±	3.3	22.2	±	3.6	**< 0.0001**	22.8	±	3.8	23.4	±	3.4	**0.024**
Arginase I	15.5	±	11.2	18.8	±	13.6	12.1	±	6.7	**< 0.0001**	15.6	±	11.3	15.4	±	11.3	0.931
L-Arginine	149.9	±	31.9	155.2	±	25.3	144.4	±	36.7	**< 0.0001**	147.0	±	33.3	152.7	±	30.3	**0.014**
NOx	24.6	±	27.2	24.8	±	24.0	24.4	±	30.2	0.258	20.8	±	23.8	28.4	±	29.8	**0.0008**
FeNO	20.5	±	13.4	20.8	±	12.7	20.2	±	14.2	0.251	21.5	±	14.5	19.5	±	12.2	0.522
FEV1%	84.6	±	9.9	86.5	±	9.1	84.7	±	10.6	0.197	87.5	±	9.5	83.8	±	10.0	**< 0.0001**
Systolic blood pressure	127.5	±	15.3	131.3	±	15.4	123.6	±	14.1	**< 0.0001**	123.7	±	13.6	131.4	±	15.9	**< 0.0001**
hs-CRP	0.70	±	1.08	0.75	±	0.99	0.65	±	1.17	**0.0004**	0.74	±	1.30	0.66	±	0.81	**0.015**
Job strain (ratio)	0.52	±	0.15	0.49	±	0.12	0.57	±	0.17	**< 0.0001**	0.52	±	0.14	0.53	±	0.17	0.809
Job demand	31.8	±	4.8	32.0	±	4.6	31.6	±	5.0	0.396	32.2	±	4.8	31.5	±	4.8	0.102
Job control	63.0	±	12.4	67.5	±	11.0	58.3	±	11.9	**< 0.0001**	63.9	±	11.8	62.0	±	12.8	0.121
Social support	22.6	±	3.3	22.8	±	3.5	22.4	±	3.0	**0.037**	22.9	±	3.6	22.3	±	3.0	0.099
Smoking habit (+/-)	116	/	252	96	/	96	30	/	156	**< 0.0001**	67	/	123	59	/	129	0.424
Exercise habit (+/-)	102	/	276	66	/	126	36	/	150	**0.0005**	57	/	133	45	/	143	0.184
Alcoholic drinker (+/-)	232	/	146	144	/	48	88	/	98	**< 0.0001**	123	/	67	109	/	79	0.177

Significant differences (P < 0.05) between sex or age are denoted in bold.

**Table 2:** Difference in variables between the company in Hiroshima and the company in Okayama.

Variables	Company 1			Company 2			P
	(n = 160)			(n = 218)			
Age	46.17	±	1.12	42.51	±	0.77	**< 0.001**
Sex (M/F)	99	/	61	93	/	125	**< 0.001**
BMI	22.32	/	0.28	22.95	/	0.24	0.315
FEV1%	89.18	±	0.73	83.15	±	0.62	**< 0.001**
Systolic blood pressure	123.25	±	1.07	131.42	±	0.91	**< 0.001**
Arginase I	20.20	±	0.78	11.61	±	0.67	**< 0.001**
L-Arginine	137.85	±	2.41	158.4	±	2.06	**< 0.001**
NOx	31.97	±	2.09	18.38	±	1.79	**< 0.001**
FeNO	18.33	±	1.07	22.06	±	0.91	**0.009**
hs-CRP	0.82	±	0.09	0.62	±	0.07	0.103
Job strain	0.54	±	0.01	0.52	±	0.01	0.14
Job demand	31.88	±	0.39	31.77	±	0.33	0.827
Job control	61.99	±	0.89	64.07	±	0.76	0.079
Social support	23.07	±	0.26	22.29	±	0.22	**0.022**
Smoking habit (+/-)	67	/	93	92	/	126	0.949
Exercise habit (+/-)	52	/	108	50	/	168	**0.039**
Alcoholic consumption (+/-)	94	/	66	138	/	80	0.369

The statistical difference of mean values of age between the two companies was analyzed by student's t test. The statistical distribution difference of sex, smoking habit, exercise habit and alcohol consumption was analyzed by chi-square test. The statitical difference of mean values of BMI, FEV1%, systolic blood pressure, arginase I, L-arginine, NOx, FeNO, hs-CRP, job strain, job demand, job control, and social support was analyzed by age- and sex-adjusted ANCOVA.

In Table 2, age and sex showed significant differences between the two companies. Therefore, age-, sex-, and company-adjusted ANCOVA was performed. FEV1%, systolic blood pressure, arginase I, L-arginine, NOx, and FeNO were significantly different. However, job strain, job demand, job control, and social support were not significantly different between the two companies. Among lifestyles factors, such as smoking habit, exercise habit, and alcohol consumption, only exercise habit was significantly different.

The median value of job strain was 0.5. By dividing the values of job strain into quartiles, differences in mean values of several NO-related parameters and other clinical parameters were evaluated by age-, sex-, and company-adjusted ANCOVA in Table 3. Significant differences were observed in arginase I and FEV1%.

**Table 3:** Age adjusted mean values of NO-related clinical variables according to quartiles of job strain.

Variables	Quartiles of job strain				
	Q1	Q2	Q3	Q4	P
Range	< 0.430556	0.430557–0.500000	0.5000001–0.5925926	> 0.5925927	
Number	97	107	79	97	
Age	43.8	43.6	43.8	43.8	
Arginase I	18.2	16.4	15.2	14.0	**0.009**
BMI	23.0	23.4	23.2	23.0	0.771
L-Arginine	148.8	148.7	148.6	152.6	0.836
NOx	23.8	24.2	25.0	30.2	0.225
FeNO	22.7	21.0	18.4	20.1	0.217
FEV1%	87.5	87.7	84.1	84.9	**0.030**
Systolic blood pressure	128.7	128.7	125.8	125.8	0.230
hs-CRP	0.70	0.71	0.71	0.75	0.991


Significant differences (P < 0.05) by age, sex and company-adjusted ANCOVA among the values of clinical parameters in quartiles of Job strain.

### Spearman’s correlation analysis between NO-related clinical parameters and job stress

**Fig 2:**
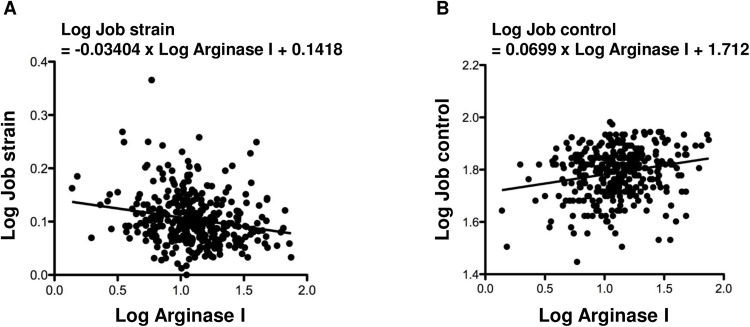
Demographic distribution between job strain and arginase I or between job control and arginase I. Log-transformed values of job strain, job control and arginase I was plotted and showed a simple regression line with regression equation. (A) job strain and arginase I; (B) job control and arginase I.

Spearman’s correlation analysis results between two variables among job strain, job demand, job control, social support, and other clinical parameters are presented in Table 4. Job strain was negatively correlated with arginase I, FEV1%, hs-CRP, and alcohol consumption. Job control was significantly correlated with BMI, arginase I, FEV1%, smoking habit, and alcohol consumption and negatively correlated with sex. Social support was significantly correlated with arginase I and FEV1% and negatively correlated with sex. The correlation between job strain and arginase I, and that between job control and arginase I were presented as demographic graphs in Fig 2A and 2B.

**Table 4:** Correlation of job strain, job demand, job control, and social support with several clinical parameters.

	Job strain (ratio)		Job demand		Job control		Social support	
	r	P	r	P	r	P	r	P
Age	0.010	0.847	-0.044	0.400	-0.071	0.174	0.035	0.503
Sex	0.297	**< 0.0001**	-0.042	0.413	-0.387	**< 0.0001**	-0.100	0.053
Company	0.027	0.607	-0.014	0.794	0.008	0.875	-0.195	**0.0002**
BMI	-0.078	0.134	0.037	0.473	0.126	**0.014**	0.015	0.775
Systolic blood pressure	-0.094	0.071	-0.054	0.298	0.095	0.066	0.004	0.941
Arginase I	-0.185	**0.0003**	-0.033	0.531	0.192	**0.0002**	0.217	**< 0.0001**
L-Arginine	-0.034	0.514	-0.063	0.222	0.029	0.571	-0.022	0.671
NOx	-0.110	**0.034**	-0.105	**0.041**	0.067	0.199	0.053	0.306
FeNO	-0.110	**0.035**	-0.022	0.671	0.053	0.309	0.061	0.242
FEV1%	-0.138	**0.007**	-0.068	0.187	0.110	**0.033**	0.159	**0.002**
hs-CRP	-0.122	**0.019**	-0.085	0.098	0.075	0.151	0.051	0.325
Smoking habit	-0.038	0.500	0.054	0.296	0.109	**0.036**	0.011	0.832
Exercise habit	-0.062	0.191	0.089	0.086	0.125	**0.015**	0.073	0.159
Alcoholic consumption	-0.178	**0.0006**	-0.014	0.792	0.203	**< 0.0001**	-0.046	0.370

Significant correlations (P < 0.05) are denoted in bold.

### Multiple regression analysis for NO-related parameters according to job stress

Multiple regression analysis showed an inverse association of arginase I with job strain, and a positive association of arginase I with job control and social support. When stratified by sex, the association of job strain with arginase I was not observed in males but was in females (Table 5).

**Table 5:** Association of job strain with arginase I in unadjusted model and adjusted model by stratification of sex.

Dependent variable	Explanatory variable	β	P
**Job strain**			
Total	Arginase I (unadjusted)	-0.410	**< 0.001**
	Arginase I (adjusted)	-0.063	**0.005**
Male	Arginase I (unadjusted)	-0.041	0.557
	Arginase I (adjusted)	-0.107	0.198
Female	Arginase I (unadjusted)	-0.196	**0.008**
	Arginase I (adjusted)	-0.172	**0.031**
**Job demand**			
Total	Arginase I (unadjusted)	-0.050	0.340
	Arginase I (adjusted)	-0.077	0.192
Male	Arginase I (unadjusted)	-0.060	0.410
	Arginase I (adjusted)	-0.115	0.166
Female	Arginase I (unadjusted)	-0.056	0.455
	Arginase I (adjusted)	-0.063	0.438
**Job control**			
Total	Arginase I (unadjusted)	0.211	**< 0.001**
	Arginase I (adjusted)	0.132	**0.016**
Male	Arginase I (unadjusted)	0.017	0.814
	Arginase I (adjusted)	0.073	0.382
Female	Arginase I (unadjusted)	0.224	**0.002**
	Arginase I (adjusted)	0.202	**0.008**
**Social support**			
Total	Arginase I (unadjusted)	0.120	**0.021**
	Arginase I (adjusted)	0.106	0.072
Male	Arginase I (unadjusted)	0.016	0.829
	Arginase I (adjusted)	0.024	0.776
Female	Arginase I (unadjusted)	0.267	**< 0.0001**
	Arginase I (adjusted)	0.244	**0.002**

β indicates standardized partial regression coefficient. Covariates included for the adjustment were company, age, BMI, L-arginine, NOx, FeNO, FEV1%, systolic blood pressure, smoking habit, exercise, and alcohol consumption. Bold represents statistical significance.

FeNO and hs-CRP were associated with job strain when covariates were not adjusted. However, when covariates such as company, age, sex, BMI, arginase I, L-arginine, NOx, FeNO or hs-CRP, FEV1%, systolic blood pressure, smoking habit, exercise habit, and alcohol consumption were adjusted, job strain was not associated with FeNO or hs-CRP (Table 6).

**Table 6:** Association of job strain (ratio) with FeNO and hs-CRP by unadjusted model and adjusted model.

Dependent variable	Explanatory variable	β	P
Job strain (ratio)	FeNO (unadjusted)	-0.178	**0.001**
	FeNO (adjusted)	-0.021	0.321
	hs-CRP (unadjusted)	0.535	**< 0.0001**
	hs-CRP (adjusted)	-0.009	0.772

β indicates standardized partial regression coefficient. Covariates included for the adjustment were company, age, sex, BMI, arginase I, L-arginine, NOx, FeNO, FEV1%, systolic blood pressure, smoking habit, exercise, and alcohol consumption. Bold represents statistical significance.

## Discussion

There is considerable evidence of a relationship between work stress and cardiovascular disease (CAD). Job strain, one of the work stress markers established by Karasek et al. [3], is also associated with CAD. CAD is preceded by the pathophysiological changes of atherosclerosis. Atherosclerosis is characterized by inflammatory damage in the vascular system. Acute or chronic stress responses may induce inflammatory responses, which are followed by the induction of acute phase proteins such as IL-1β, IL-6, and C-reactive protein [25, 26] Job strain has been associated with inflammation-related proteins including IL-8 and MCP-1 [27, 28]. Moreover, CRP was found to be associated with job strain, which is mediated by physical activity, in the MONICA/KORA study [29]. Therefore, in the present study, we considered it a prerequisite to investigate the relationship between job strain and CRP in order to clarify that between job strain and arginase I. However, in this study, job strain was inversely associated with arginase I. Social support was positively associated with arginase I. Previously, we showed a high serum level of arginase I in asthma and the correlation of arginase I with hs-CRP [30], as well as the association of arginase I with oxidative stress [17, 18]. Oxidative stress is associated with depression [31–34]. Several studies showed that increased job demands were associated with depressive symptoms and distress [35, 36]. Moreover, job strain was strongly associated with depression. From this previous evidence, it is speculated that job strain or job demand is associated with oxidative stress and up-regulation of arginase I. On the contrary, the evidence of this study did not coincide with previous studies since job strain was negatively associated with arginase I. However, job strain here was the ratio obtained by dividing the score of job demand by that of job control.

Arginase I was not considered to be associated with job demand, but was with job control and social support. That is, the negative association of arginase I with job strain was not dependent on job demand but was dependent on job control. Moreover, when the relationships between job strain, job demand, job control, social support, and arginase I were evaluated by the stratification of sex and company, correlations were observed between arginase I and job strain, job control, and social support in females, and between arginase I and job control in both companies. The strength of the relationship between job strain and arginase I was weaker in company 2 than in company 1 (S1 Table). Regarding sexual differences in the relationship between job strain and ambulatory blood pressure, there was no inconsistency [37–39]. However, ambulatory blood pressure was greater in men and women with low job control, in which job demand and job strain were only weakly related to blood pressure [40]. In the Nurse Health Study, a large prospective cohort of 35,000 female nurses, job strain was not associated with incident coronary heart disease in a 4-year follow-up; however, job insecurity was related to the risk of non-fatal myocardial insufficiency over 2 years [41]. Therefore, since there is evidence to support vascular endothelial factor being associated with not only job strain, but also job control, serum arginase I levels may also be associated with job control and social support, while arginase I may be a psychologically relaxing factor through mechanisms that have not yet been elucidated.

In this study, job strain was negatively correlated with hs-CRP and FeNO. However, regression analysis between job strain and hs-CRP or FeNO did not show significant association (Table 6). In a Korean study of 155 male bank workers, job stress was associated with hs-CRP [42]. However, job strain was not correlated with hs-CRP [29, 43]. Therefore, the lack of association of job strain, job demand, job control and social support with hs-CRP in this study (S2 Table) may be dependent on the difference in covariates used in each study.

As for FeNO, the association of FeNO with depression was observed in several studies [7, 9, 10, 44]. Depression was associated with high levels of CRP and low levels of FeNO [10]. In asthma, acute stress induced up-regulation of FeNO, and depressive mood decreased FeNO [44]. However, another study showed the increased association of depressive mood with FeNO. There was no evidence to show the association of job strain with FeNO. High job strain is not a pathological state, but may be a precondition of depression.

Although the results obtained here are significant, several limitations in the study need to be noted. First, since the sample size was small, the analysis power decreased when stratification was applied. Second, casual relationships could not be determined because this study was a cross-sectional study. Third, some reporting bias may have been introduced because the information on job stress was obtained via self-reported questionnaires. Fourth, to explore the relationship between job strain with arginase I in females, information about menstrual cycles is needed. Fifth, for an analysis of job stress, information about clerical staff or physical labor is needed.

The inverse association of job strain with arginase I and the positive association of job strain with job control and social support suggest that serum arginase I may be a useful biomarker for the screening of job strain in workers. However, to verify this hypothesis, an analysis of more workers is needed.

## Supporting information

S1 Table Association of job strain with arginase I in unadjusted model and adjusted model by stratification of company. (XLSX)

S2 Table Association of job strain (ratio), job demand, job control and social support with hs-CRP by unadjusted model and adjusted model. (XLSX)
